# Validation of a new test that assesses functional performance of the upper extremity and neck (FIT-HaNSA) in patients with shoulder pathology

**DOI:** 10.1186/1471-2474-8-42

**Published:** 2007-05-17

**Authors:** Joy C MacDermid, Myriam Ghobrial, Karine Badra Quirion, Mélanie St-Amour, Tanya Tsui, Dave Humphreys, John McCluskie, Eddy Shewayhat, Vickie Galea

**Affiliations:** 1 Clinical Research Lab, Hand and Upper Limb Centre, St. Joseph's Health Centre, London, Ontario N6A 4L6, Canada; 2 School of Rehabilitation Science, McMaster University, Hamilton, Ontario L8S 1C7, Canada; 3 Heart Lake Sports Medicine & Orthopedic Clinic, Brampton, Ontario L6Z 1Y4, Canada; 4 Fowler Kennedy Sport Medicine Clinic, London, Ontario N6A 3N7, Canada; 5 Hamilton General Hospital, Hamilton Health Sciences, Hamilton, Ontario L8L 2X2, Canada; 6 Sarnia Community Care Physiotherapy, Sarnia, Ontario N7T 7W5, Canada; 7 Université de Sherbrooke, Sherbrooke, Quebec J1K 2R1, Canada

## Abstract

**Background:**

There is a lack of standardized tests that assess functional performance for sustained upper extremity activity. This study describes development of a new test for measuring functional performance of the upper extremity and neck and assesses reliability and concurrent validity in patients with shoulder pathology.

**Methods:**

A series of developmental tests were conducted to develop a protocol for assessing upper extremity tasks that required multi-level movement and sustained elevation. Kinematics of movement were investigated to inform subtask structure. Tasks and test composition were refined to fit clinical applicability criteria and pilot tested on 5 patients awaiting surgery for shoulder impingement and age-sex matched controls. Test-retest reliability was assessed on 10 subjects. Then a cohort of patients with mild to moderate (n = 17) shoulder pathology and 19 controls (17 were age-sex matched to patients) were tested to further validate the Functional Impairment Test-Hand, and Neck/Shoulder/Arm (FIT-HaNSA) by comparing it to self-reported function and measured strength. The FIT-HaNSA, DASH and SPADI were tested on a single occasion. Impairments in isometric strength were measured using hand-held dynamometry. Discriminative validity was determined by comparing scores to those of age-sex matched controls (n = 34), using ANOVA. Pearson correlations between outcome measures (n = 41) were examined to establish criterion and convergent validity.

**Results:**

A test protocol based on three five-minute subtasks, each either comprised of moving objects to waist-height shelves, eye-level shelves, or sustained manipulation of overhead nuts/bolts, was developed. Test scores for the latter 2 subtasks (or total scores) were different between controls as compared to either surgical-list patients with shoulder impingement or a variety of milder shoulder pathologies (p < 0.01). Test 1 correlated the highest with the DASH (r = -0.83), whereas Test 2 correlated highest with the SPADI (r = -0.76).

**Conclusion:**

Initial data suggest the FIT-HaNSA provides valid assessment of impaired functional performance in patients with shoulder pathology. It discriminates between patients and controls, is related to self-reported function, and yet provides distinct information. Longitudinal testing is warranted.

## Background

Given that shoulder pathology has a substantial impact on quality of life [1-5] that is lasting [6,7], and only 59% of shoulder complaints resolve within 12 months [8], reliable and valid methods of determining risk, severity, and outcome for these disorders are imperative.

Strength and stability must co-exist to insure optimal shoulder function. Given the key role of muscles in establishing shoulder stability, mobility, and function, it is not surprising that the strength of specific muscle groups is typically viewed as a key outcome measure when evaluating shoulder conditions. Quantitative measures of isometric [4,9-12] and isokinetic [4,11-23] strength have been described to dictate the functionality of muscles in different muscle actions. The importance of muscle strength has been confirmed in that shoulder strength has been shown to be related to general health status in persons with shoulder pathology [4]. Range of motion (ROM) [24] is also frequently reported in shoulder studies, although its relationship to function has been less clearly defined [25]. Shoulder functionality requires coordinated, sustained muscle activity that both maintains sufficient proximal control and also allows a wide arc of pain-free movement for completion of tasks of daily life. Thus, it is not unexpected that isolated physical impairments like muscle strength or ROM deficits have demonstrated small to moderate correlation to function. However, this suggests that better understanding of function requires specific functional tests.

One approach to "functional outcomes" has been the use of patient-self report. Pain is a primary symptom in most shoulder disorders. In fact, shoulder pain has been a primary focus of systematic reviews looking at diagnosis [26], prognosis [6], and treatment effectiveness [27,28]. These reviews have emphasized the need for validated outcome measures to improve the validity of clinical trials addressing shoulder pain [27]. A number of self-report measures have been designed to assess shoulder pain and disability from the patient's perspective. These include disease-specific quality-of-life measures [29,30], joint-specific pain/disability measures [31], and regional measures [32,33]. Self-report measures have the advantage of being patient-centered and relatively easy and inexpensive to apply. They provide an important perspective on patient status. However, studies conducted in different upper extremity clinical populations [34-37], and specifically, shoulder problems [4,38,39] agree that the relationship between self-reported disability and actual physical impairment or functional performance is moderate [40]; thus, they cannot be used as surrogates. This suggests the need for a valid structured test of functional performance of the upper extremity that incorporates shoulder and neck.

While a variety of hand function or dexterity tests have been described [41-46], most tasks involve minimal shoulder movement, nor were they developed or validated to assess patients across a spectrum of shoulder conditions. An exception is the test developed by Hughes et al. [47], the *Simple Shoulder Endurance Test*. Unfortunately, this test assesses only one task and demonstrated limited reliability. There are also reports of "endurance" or "fatigue" protocols that can be used on isokinetic dynamometers [48-51], although most last less than 2 minutes, suggesting they do not assess the physiologic pathways used in most sustained work or functional activity.

Therefore, our aim was to (i) develop a test protocol that requires coordinated movement and positioning of the upper extremity and neck across different tasks that simulate elements of functional activity, and (ii) test its validity and reliability in a first phase diagnostic study.

## Methods

### Study overview

1. Phase 1 – Protocol Development

2. Phase 2 – Psychometric Evaluation: (Preliminary) Reliability Testing

3. Phase 3 – Psychometric Evaluation: Validation of FIT-HaNSA in patients with mild-moderate shoulder pathology

### Protocol development

The test was developed to address tasks of functional relevance that challenged the upper quarter, including shoulder/neck, with content relevance determined by both clinical experience and kinematic analyses. Preliminary task definition that described the nature of the tasks was completed in the Human Movement Laboratory at McMaster University. The kinematics of the upper extremity during a reach and grasp task were evaluated in a series of studies evaluating motor control of the upper extremity and neck (described below). Literature describing the kinematics of shoulder function tests [47], hand dexterity tests [41,42,45,46,52-54], the first author's experience in test development, and problems reported by patients with shoulder conditions were also used to develop the preliminary protocol. The following criteria for relevance and clinical utility were established as necessary components during test development:

1. have a variety of subtasks that included gross motor and sustained movements of the upper extremity and neck including shoulder/neck/elbow.

2. should be relevant to a wide spectrum of disorders by having subtasks that would be completed by subjects with severe pathology as well as those with mild pathology.

3. should be completed using readily available commercial equipment or be easily constructed from routine materials.

4. should be easily scored.

5. should assess endurance to sustained "functional" activity in a standardized format, i.e., > 5 minutes of muscle activity, but be reasonable to perform in a clinical environment, i.e., < 20 minutes using standardized methods.

**Figure 1:**
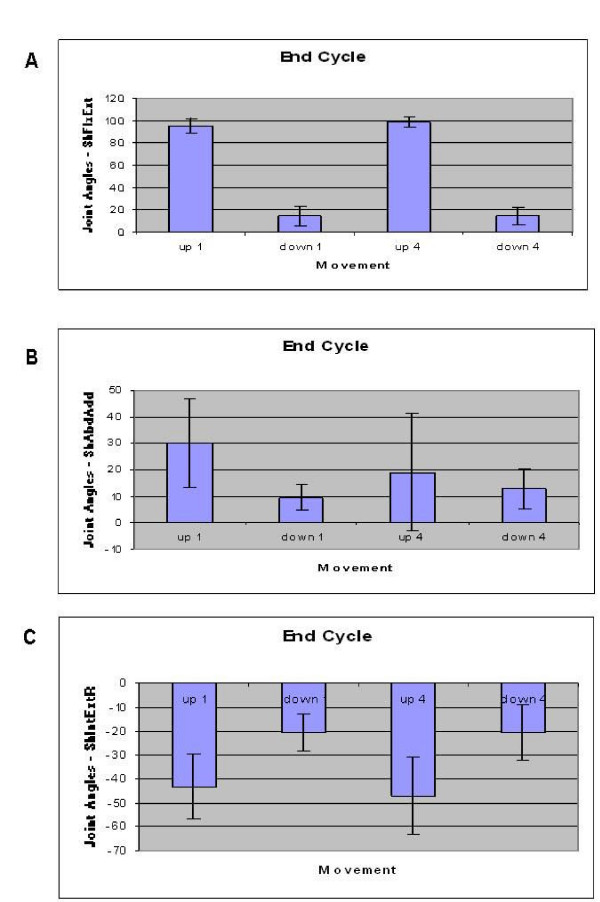
Shoulder ROM during repetitive high height task. Values acquired from development studies. A – Shoulder Flexion/Extension; B – Shoulder Abduction/Adduction; C – Shoulder Internal/External Rotation.

Task definition was conducted on 12 volunteers with no neck, shoulder, arm, nor hand pathology. The age range for this set of subjects was 19–21 years since all were undergraduate university students. Segmental 3-dimensional kinematic behaviour was acquired using a magnetically-based motion tracker (Polhemus Systems, Skills Technology). We acquired the 3-dimensional position and orientation data through electromagnetic sensors placed on the neck (upper trunk), upper arm, forearm, and hand segments to represent the shoulder, elbow, and wrist joints. The joint angular displacement time series were most informative to the "challenge" required for patients with various neck, shoulder, and arm pathologies to perform. As an example, the ranges of motion required for the "Repetitive High Height" task were at least 120° of shoulder flexion, 25° of shoulder abduction, and at least 30° of external rotation (See Figure 1). These ranges had to be sustained throughout the testing period (3 minutes) at a metronome speed of 60 beats per minute.

Potential "pilot" tasks were reviewed by the first author and reconfigured to fit with the criteria for relevance and clinical utility. During the next phase of test development, knowledgeable physiotherapists and physiotherapy students reviewed potential test components. Issues on spectrum of testing, test components, test procedures, scoring, and the potential influence of different pathologies were discussed to insure that the test components would be feasible and useful for clinical assessment of patients. Iterative test development sessions were conducted to refine this "alpha protocol". Minor changes were made to increase the difficulty of the tasks and enhance their clinical application.

### The test protocol

**Figure 2:**
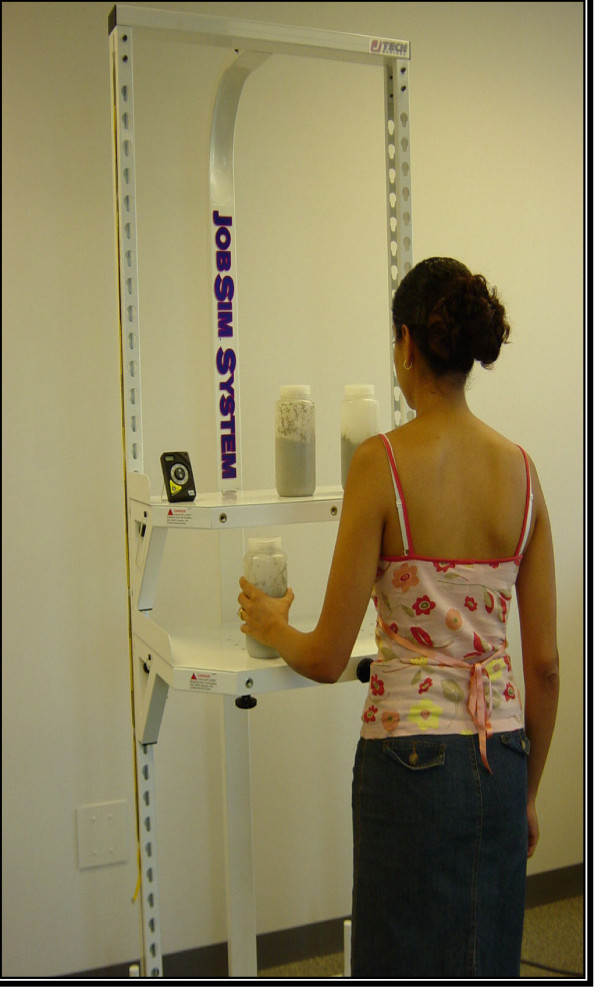
Task 1, "Waist-Up".

**Figure 3:**
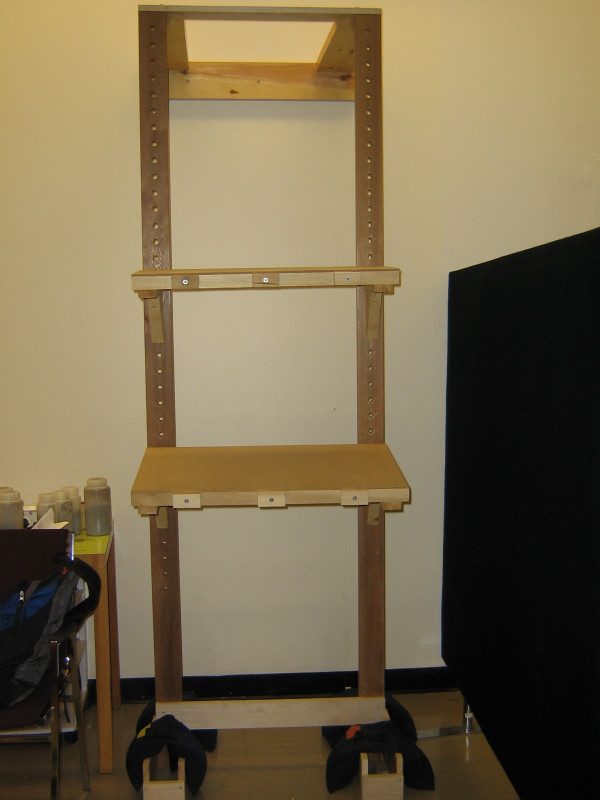
Wooden Version of the FIT-HaNSA.

The final functional assessment protocol is called the Functional Impairment Test-Hand and Neck/Shoulder/Arm (FIT-HaNSA) and consists of a battery of three tasks that simulate daily activities of lifting and sustained overhead work in the household or workplace. FIT-HaNSA was designed to test the endurance of the shoulder, with the expectation that the difficulty of the sustained tasks would distinguish between individuals with varying degrees of shoulder function with the relative task difficulty depending on the nature of the underlying shoulder pathology. The JobSim (JTech Medical, Salt Lake City, USA) was used for all tests (Figure 2); however, the test could also be reproduced with commercial shelving/hardware or custom-made materials. In fact, a wooden version is currently in use in our Human Movement Laboratory (Figure 3) (see additional file 1: The FIT-HaNSA Protocol for a detailed description) for studies which require the use of electromagnetic devices.

The test consists of 3 subtasks, the first being the simplest to perform for the majority of individuals. A test manual is provided as a supplementary file (See additional file 1: The FIT-HaNSA Protocol). Each task can be continued for up to 5 minutes, but is terminated based on the following stopping rules:

1. The subject stops or states it is too painful to continue.

2. The subject is severely off pacing (provided by a metronome) to the extent that they are unable to complete one repetition of the movement within 2 beats of the metronome (for 5 successive repetitions).

3. The subject substitutes using trunk/whole body movement and cannot correct with feedback for 5 successive repetitions of the task.

4. The examiner believes the subject is at risk of injury or adverse events if the test were to continue.

In the first task ("waist-up"), a shelf was placed at waist level and a second shelf was placed 25 cm above it; three 1-kg containers were placed 10 cm apart on the lower shelf (Figure 2). Using the affected arm, the patient would lift the 3 containers, one at a time, from one shelf to the other at a speed of 60 beats per minute, controlled by a metronome. The subjects and controls were instructed to do the test until 5 minutes have elapsed or they feel unable to continue. The time to complete the task was measured by a stopwatch.

**Figure 4:**
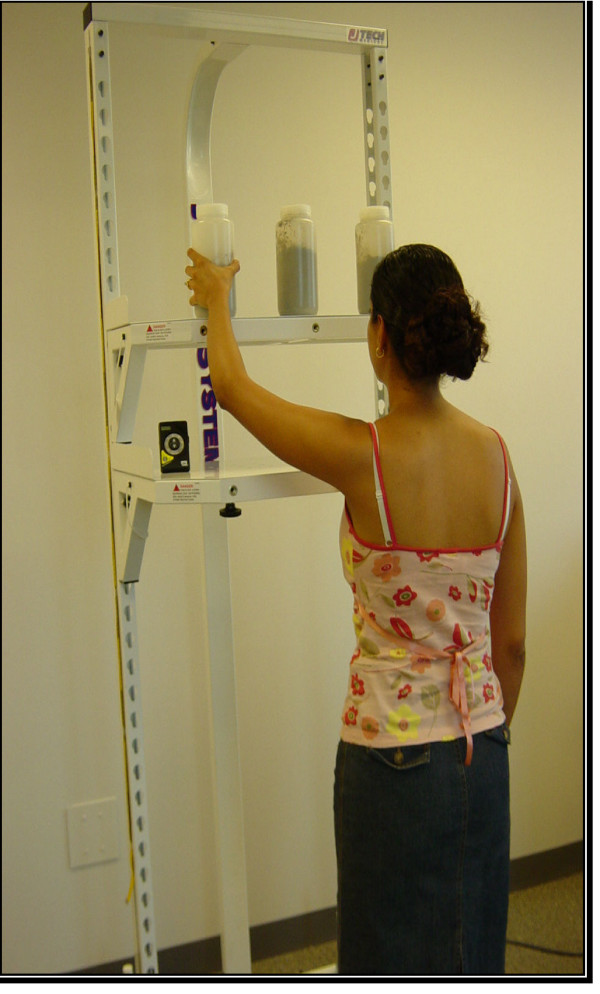
Task 2, "Eye-Down".

In the second task ("eye-down"), the shelves were adjusted so that one shelf was placed at the subject's eye level and the second shelf was placed 25 cm below it (Figure 4). The patients were again instructed to use their affected arm to lift the three containers between the shelves at a speed of 60 beats per minute. The same stopping protocol was used for task 2.

**Figure 5:**
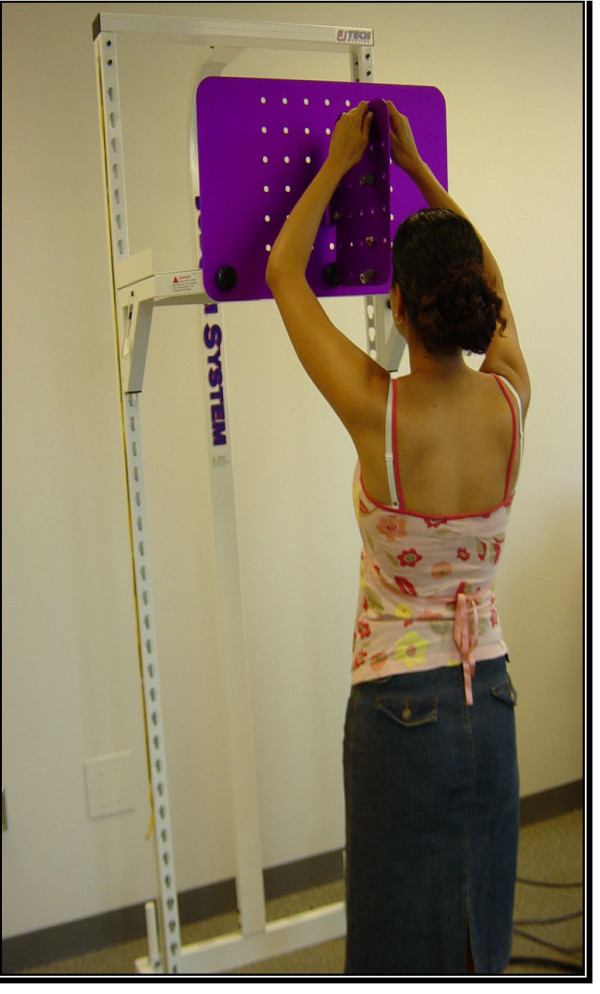
Task 3, "Overhead Work".

In task 3 ("overhead work"), a shelf was placed at the subject's eye level with an attachable plate, perpendicular to the shelf, projecting out toward the subject (Figure 5). Patients were instructed to use their affected arm to repeatedly screw and unscrew bolts in a pattern (the bolt in notch 1 (top) moves down to notch 2 (middle); the bolt in notch 3 (bottom) moves up to notch 1; the bolt in notch 2 moves down to notch 3) into the plate. The same stopping protocol was used for task 3. Arms were not dropped during testing.

There was an approximately 30-second rest between the tests as the shelves were adjusted for the different tasks.

### Psychometric objectives

1. To develop a test protocol that could be completed by most subjects without shoulder pathology, but sensitive to detecting functional impairment across a spectrum of severity (mild to severe).

2. To describe the central tendency and variability of this test in subjects with shoulder pathology as compared to age-sex matched controls.

3. To provide preliminary estimates of reliability.

4. To determine the concurrent convergent validity by comparing the new test to indicators of impairment in patients with shoulder disorders-including self-reported function and isometric muscle strength.

5. To determine the construct validity of the new test by assessing whether the following constructed hypotheses were supported:

a. Subjects with mild-moderate impingement would be different from controls.

b. Subjects with impingement would have minimal difficulty with a low level reach task with light weight, but more difficulty with tasks in impinged positions.

### Participants

#### Group 1 – Early validation in high severity subgroup

Subjects (n = 5) were recruited from the surgical wait-list of a single orthopedic surgeon. Patients with a diagnosis of shoulder impingement unresponsive to conservative management but without complicating co-morbidity were tested on a single occasion. Subjects were identified as having shoulder impingement on the basis of a physical examination by a shoulder surgeon which was further confirmed on the test occasion by a positive Hawkins test [55-57]. Control subjects were sex- and age-matched (± 5 years). The age range of subjects was 25–76. The Hamilton Health Sciences Research Ethics Board approved the protocol.

#### Group 2 – Reliability testing

A subgroup of patient and controls (n = 10) who agreed to duplicate testing were evaluated on two occasions; Mean age = 39 ± 14; 2 M, 8 F (3 patients, 7 controls).

#### Group 3 – Validation in mild-moderate shoulder pathology

Thirty-six subjects were recruited in total; patients with shoulder pain (n = 17) were recruited from the sports medicine/staff physiotherapy clinic at McMaster University (see Table 1). The patients' shoulder pathologies included: bicipital tendonitis, bursitis, chronic subluxation, capsulitis, impingement, instability, and surgical repair of the labrum. Shoulder patients were not excluded if they also reported hand, elbow, or neck pathology. The control group consisted of nineteen subjects with no shoulder pathology as indicated by self-report (direct question and SPADI scores); 17 were sex- and age-matched (± 5 years) to tested patients. The mean age of the experimental and control subjects was 32 years (range = 20–62 years). The McMaster University Ethics Board approved the study and informed consent was obtained from all subjects prior to testing.

### Comparative measures

All subjects completed the Disabilities of the Arm, Shoulder, Hand (DASH) [33,58], and the Shoulder Pain and Disability Index (SPADI) [31,59,60]. In part 3 of the study, isometric shoulder abduction and flexion strength for the test shoulder were measured using the Lafayette Manual Muscle Tester (Lafayette Instrument Company, Lafayette, USA). Isometric strength testing was performed in a stable seated position with the arm in neutral (3 repetitions averaged).

## Analyses

During the developmental work (phase 1), the tests were modified so that the instructions were standardized, the break between subtasks (to further emphasize endurance) was decreased, and the motions performed were clarified. In phase 2, Intra-class Correlation Coefficients (2,1) [61] were used to describe test-retest reliability. For all data subgroups, scale scores and variability, descriptive statistics of the task, and test performance were computed. For phase 3 validity analyses, differences were determined between subgroups by using ANOVA. Pearson correlations were used to compare strength and self-reported function measures to FIT-HaNSA scores. All statistical analyses were conducted in SPSS 14.0.

## Results

### Early validation (severe pathology)

Data provided by group 1 provided preliminary data (wide variation/small sample size) that compared subjects with severe shoulder impingement to age-matched controls with no shoulder pathologies. Patient stopped the subtests for a combination of discomfort and fatigue. The mean times for tasks 1, 2, and 3 for those with shoulder impingement were 179 s, 117 s, and 151 s, respectively. Controls completed all subtasks except for Task 2 in 1 control, providing means of 300 s, 286 s, and 300 s, respectively, for those with no shoulder pathologies. Despite the small sample, the mean times for tasks 1, 2, 3 for subjects with shoulder impingement were significantly shorter than the age-sex matched control group (p = 0.041). The percentage of task completion (mean time spent on all three tests/300 s) for subjects 1, 2, 3, 4, and 5 were 33%, 12%, 22%, 86%, and 95%, respectively, when using 300 s as the referent standard. The Pearson correlation coefficients between FIT-HaNSA and the SPADI or DASH were -0.85 and -0.94, respectively.

### Reliability

ICCs (2,1) (95% confidence intervals) for test-retest reliability of scores for Group 2 were:

1. Test 1: 0.97 (0.84–0.99)

2. Test 2: 0.98 (0.94–1.00)

3. Task 3: 0.96 (0.85–0.99)

4. 3-Task Mean Score: 0.98 (0.90–0.99)

### Validation in mild-moderate pathology

**Figure 6:**
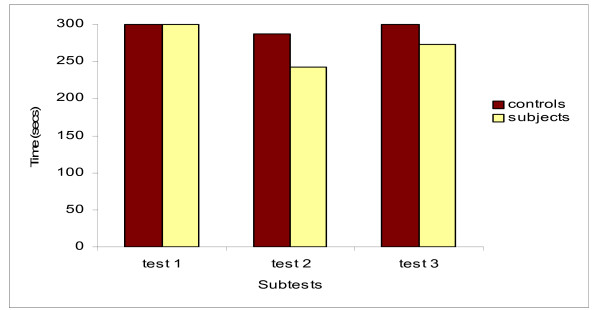
Comparison of Mean Group Times. This figure shows FIT-HaNSA times for shoulder patients versus controls. Significant differences were observed for Task 2 and Task 3. All subjects performed 5 full minutes of Task 1.

In group 3, the patients' mean times with 95% confidence intervals in brackets for tasks 1, 2, and 3 were 300 s, 246 s (208–284), and 275 s (249–300), respectively, whereas the controls' mean times were 300 s, 290 s (278–300), and 300 s, respectively (Table 1). The patients' total mean time was 274 s (256–291), compared to the controls' 297 s (293–300). There was no significant difference between patients and controls for isometric strength. Significant differences were found between the groups for the DASH (p = 0.005), the SPADI (p = 0.002), and the average total time on FIT-HaNSA (p = 0.008). Test 2 demonstrated the largest difference between patients and controls (p = 0.021). Test 3 was also statistically different between the groups (p = 0.044) (Figure 6). Test 1 correlated the highest with the DASH (0.01 level, r = -0.83), whereas Test 2 correlated highest with SPADI (0.01 level, r = -0.76) (Table 2). The correlations between isometric strength scores and the DASH or SPADI were non-significant.

## Discussion

This study describes the development of a new functional performance test for shoulder pain patients and provides preliminary results for its validity and reliability. Discriminative validity and construct/criterion validity have been supported in two groups of patients. In a small group of more severely affected patients with uncomplicated impingement awaiting surgery, the differences in scores were profound and statistically significant despite a very small sample size. In patients with milder conditions who were involved in active rehabilitation, the differences were less extreme but also statistically significant. It is noteworthy that isometric strength scores were not affected in this same group, suggesting that the FIT-HaNSA was better able to detect the physical impairments associated with these milder pathologies (i.e., was more discriminative). Preliminary reliability testing of a small mixed group was encouraging with all reliability coefficients exceeding 0.95.

Our test was designed to be assessed in a spectrum of shoulder patients. Task 1 is the first and easiest to perform. We anticipated that subjects with severe shoulder pathology like total shoulder arthoplasty, severe osteoarthritis, or with proximal humeral fractures might have difficulty with this task. However, we did not test that assumption. Clearly, this task contributes to the overall endurance load placed the shoulder, but was included in the protocol to insure that floor effects were not a problem. It will be important to determine whether this subtask detects a spectrum of capability in patients with more severe shoulder conditions.

We recognize limitations in specific examination tests and in the process of making definitive diagnoses in shoulder disorders, particularly for "shoulder impingement" which is really a symptom not a diagnosis. Nevertheless, "impingement" is one of the most common problems affecting the shoulder [62,63] and its presence in our subjects was confirmed by expert orthopedic surgeons and physiotherapists. Task 2, which involves repetitive movement into a relatively impinged position during a grasp and place activity, was anticipated to be challenging for these patients. In our developmental work with patients who have severe impingement, this was very evident as task 2 was grossly limited and more limited than other tasks. Our second validation group included a spectrum of patients including a number with impingement and again, Task 2 was the most challenging. Due to the nature of the test protocol used in Phase 2, we had longer breaks between subtests and this may also have contributed to better performance on subsequent tasks. We suggest that the appropriate rest between subtasks should be the time required to adjust shelves (less than 30 seconds).

We had anticipated that Task 3, where sustained overhead work is performed, might be most challenging for patients with shoulder/neck pain, particularly if associated with radiculopathy or for other conditions involving neural structures such as thoracic outlet syndrome. Again, these subgroups were not tested so this assumption is also not yet validated. However, the range of tasks included in FIT-HaNSA is consistent with a range of functional activities that would be affected in shoulder disorders.

There are a number of variations between FIT-HaNSA and the other shoulder functional test reported in the literature. The "Simple Shoulder Endurance Test" reported by Hughes [47] consists of a single activity (screwing bolts) motion that required shoulder stability at 45° of forward flexion, but not movement of the shoulder. The task was performed with increasing weight every 2 minutes and the average amount of time to test termination was 413 seconds. This is similar to our 300-second subtask time, but there are advantages to our staged 3-level subtask approach as compared to the single task protocol described above. One advantage of FIT-HaNSA is that it tests multiple functions of the shoulder and neck, in particular, assessing both stabilization and movement. We used a standing position as the sitting and belted position does not represent the way most functional tasks would be performed. A further advantage is that functional endurance is assessed over a longer period of time which may be more reflective of actual functional performance.

We were encouraged by a number of findings. The discriminative validity of FIT-HaNSA was greater than demonstrated by isometric strength testing with a hand-held dynamometer. This suggests that the test is providing useful information about the physical status in mild to moderate shoulder disorders. Reliability scores, while based on a small and broadly-based sample, were excellent. We anticipate that reliability coefficients may be lower when the test is performed in more homogeneous populations, as a more variable population favors achieving high ICCs. Nevertheless, favourable preliminary reliability evaluation indicates that these investigations of test behaviour in different subpopulations are indicated.

While the reliability, discriminative validity, and score ranges demonstrated in this study may imply an ability for the test to evaluate change over time, our study was cross-sectional and, thus, we are unable to make such conclusions. We acknowledge these results are preliminary and purposively wish to share the protocol at this early stage to allow others to implement it and potentially to participate in independent validation. While the numbers reported in this study are small, 2 years of development and piloting was conducted to devise and evaluate a protocol that is ready for widespread clinical field testing. It is important that while we proceed with additional validation, independent authors have the opportunity to do as well. This should include different types of upper extremity/neck pathology, across different age/sex subgroups and test environments. Substantial gaps in knowledge about the psychometrics of this test remain, notably the longitudinal validity, including responsiveness. This study reports the iterative development process and early positive psychometrics properties of a new functional performance test.

## Conclusion

This study provides preliminary support for the validity of the FIT-HaNSA in clinical evaluation of shoulder disability in that it is tolerable for most people without pathology, completed in 20 minutes, has acceptable test-retest reliability, and discriminates between controls and patients in both severe and mild to moderate shoulder pathology. It provides different information than self-reported function and, thus, provides an alternative means to assess functional status or progression of functional ability over time. Future studies should focus on evaluating its responsiveness in longitudinal studies and its psychometric properties across different upper extremity pathologies.

## Competing interests

The author(s) declare that they have no competing interests.

## Authors' contributions

VG has conducted work on kinematics of the upper limb and discussed this with JM when FIT-HaNSA was being developed. JM developed the preliminary clinical test protocol and worked with 2 research groups to refine the test protocol and validate it. The first group (DH, JM, ES) helped with test protocol development and tested patients awaiting surgery for surgery for impingement repair, and the second study group (MG, KBQ, MS, TT) evaluated FIT-HaNSA in patients with a spectrum of milder shoulder pathologies. JM conducted statistical analyses and drafted the manuscript. All authors approved the final study protocol, contributed to interpretation of the study results, and participated in revisions of the manuscript. All authors read and approved the final manuscript.

## Pre-publication history

The pre-publication history for this paper can be accessed here:



## Supplementary Material

Additional File 1 The FIT-HaNSA Protocol Click here for file

## Figures and Tables

**Table 1:** Demographics of Validation Study Participants

	**Controls (n = 19)**	**Subjects (n = 17)**
Age (yrs)	32 (12)	32 (12)
Sex	11 females, 8 males	9 females, 8 males
Flexion (lbs of F)	34.11 (14.67)	30.46 (14.30)
Abduction (lbs of F)	33.75 (14.20)	30.55 (13.41)
DASH	1.10 (1.57)	10.25 (12.57)
SPADI	1.26 (2.40)	16.41 (18.60)
Test 1 (secs)	300 (0)	300 (0)
Test 2 (secs)	290 (25)	246 (74)
Test 3 (secs)	300 (0)	275 (51)
Ave. total time (secs)	297 (8)	274 (34)

Mean (Standard Deviation)

**Table 2:** Pearson correlations between the FIT-HaNSA tasks and strength or self-report scores

	**Task 1**	**Task 2**	**Task 3**
**Flexion strength**	--	0.29 (n = 36)	0.15 (n = 36)
**Abduction strength**	--	0.27 (n = 36)	0.12 (n = 36)
**DASH**	-0.83** (n = 41)	-0.82** (n = 41)	-0.73** (n = 41)
**SPADI**	-0.74** (n = 41)	-0.76** (n = 41)	-0.67** (n = 41)

** Correlation is significant at the 0.01 level (2-tailed).

Note: The correlations between task 1 and strength could not be computed because all subjects completed the 300-seconds task.

The correlations between the tasks and self-report scores included the 5 patients from the development phase (part 1).
