# Supplementary material for: Validation of a new test that assesses functional performance of the upper extremity and neck (FIT-HaNSA) in patients with shoulder pathology
Source: BMC Musculoskelet Disord. 2007 May 17;8:42. doi: 10.1186/1471-2474-8-42 (PMC1892014; doi:10.1186/1471-2474-8-42)
Supplement: Additional File 1 — The FIT-HaNSA Protocol [file 1471-2474-8-42-S1.doc]

**The Functional Impairment Test-Head, and Neck/Shoulder/Arm**

**(FIT-HaNSA) Protocol**

Joy MacDermid

School of Rehabilitation Science, McMaster University, Hamilton, Ontario, Canada

Clinical Research Lab, Hand and Upper Limb Centre, St. Joseph’s Health Centre, London, Ontario, Canada

E-mail: macderj@mcmaster.ca or jmacderm@uwo.ca

**Test Purpose: To provide a brief measure of functional performance of the upper limb while performing multi-level tasks that require grip/manipulation of the hand, elbow/shoulder reaching, sustained overhead work, and sustained positioning with a particular emphasis on assessing the limitations in functional performance attributable to shoulder/neck disorders.**

**Test Equipment**

- The JobSim System (JTech Medical, Salt Lake City, USA) can be used for all FIT-HaNSA tests.
- The test can also be reproduced with self-made materials using instructions in Appendix 1.

**Set-Up with JTech Equipment**

*Test 1 – “Waist-up”*


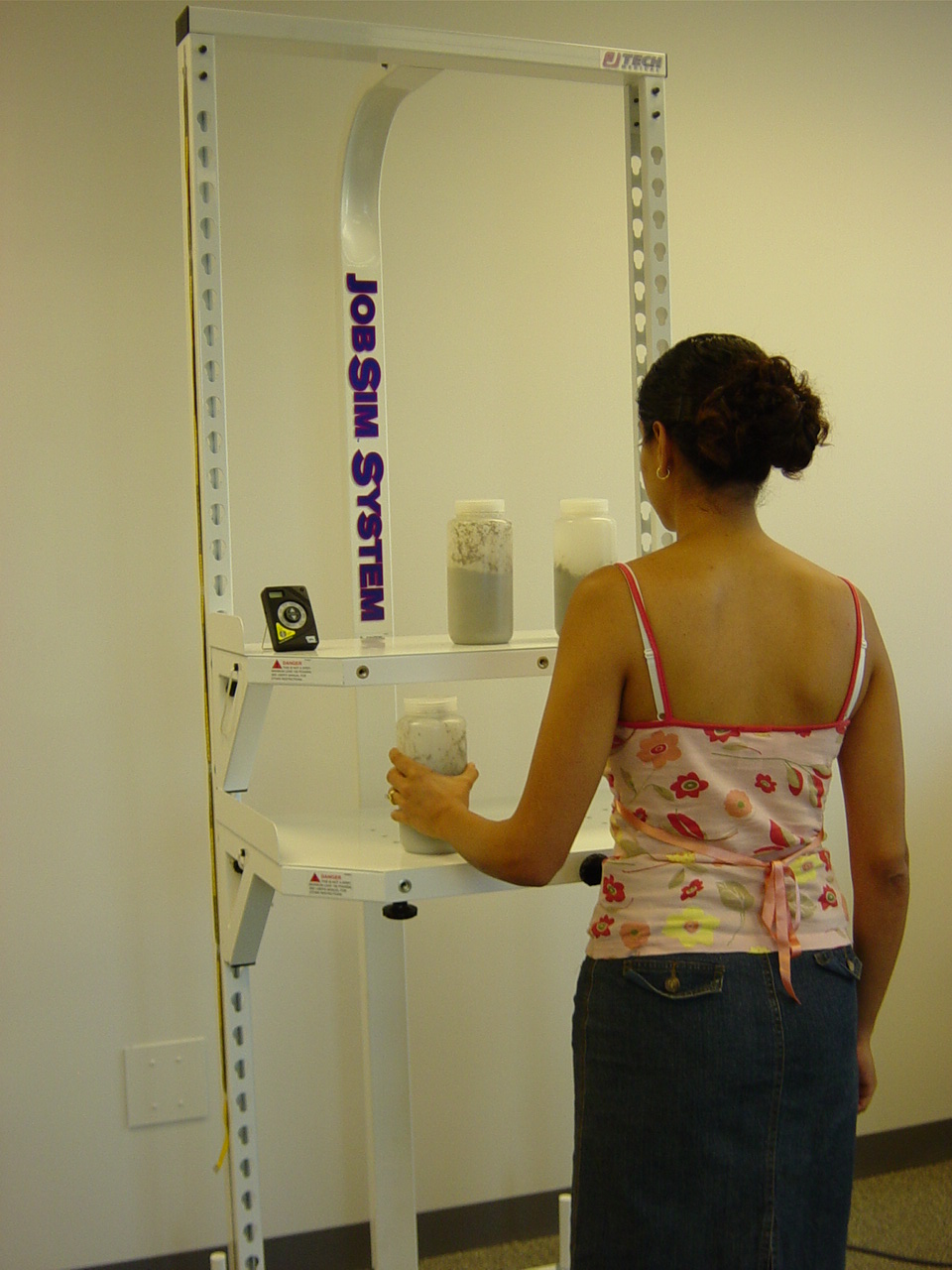


One shelf is placed at the subject’s waist level and a second shelf is placed 25 cm above it. The three 1-kg containers are placed 10 cm apart, in line with the screws on the upper shelf, on the lower shelf.

*Test 2 – “Eye-down”*


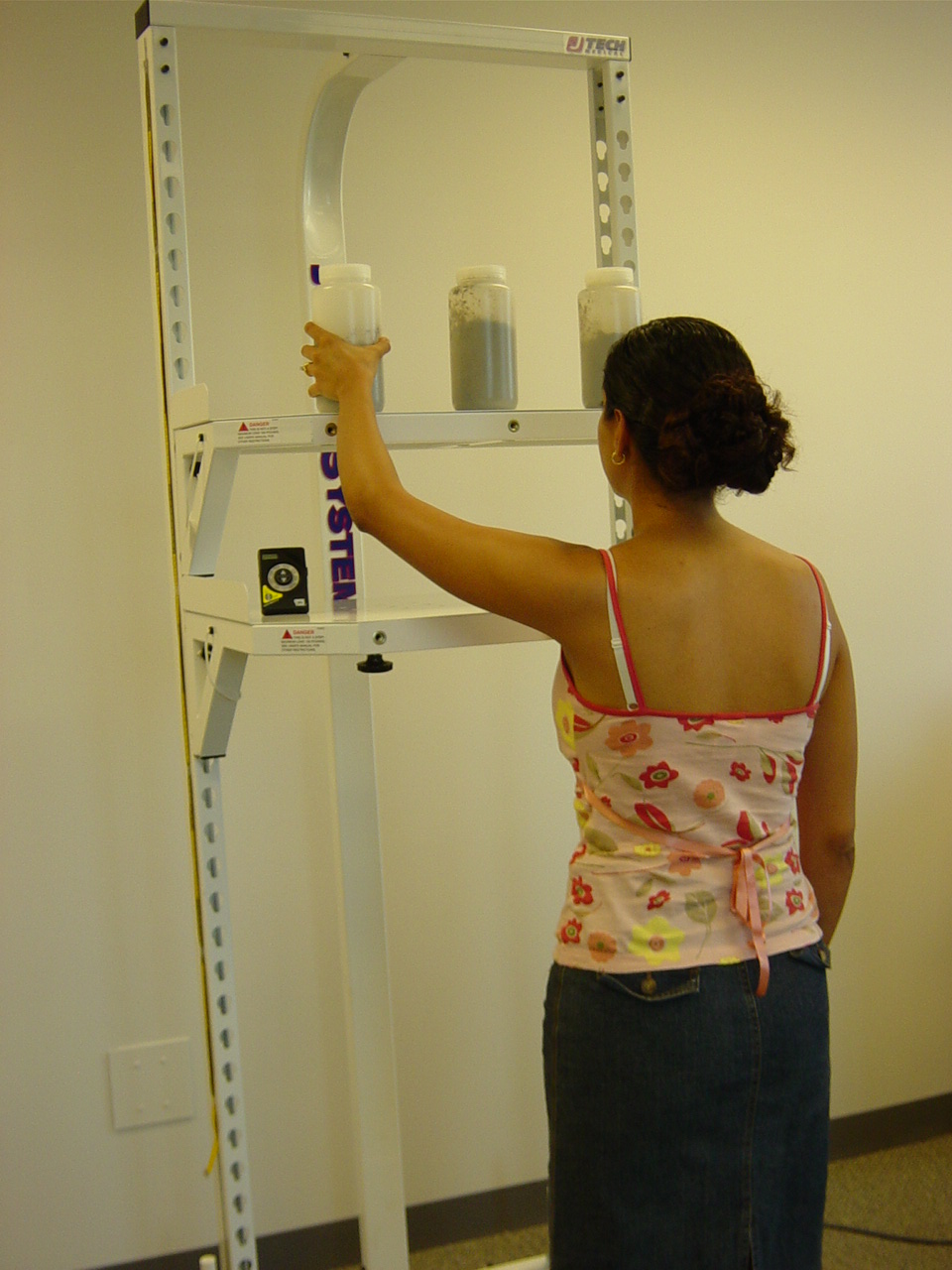


One shelf is placed at the subject’s eye level and a second shelf is placed 25 cm below it. The three 1-kg containers are placed 10 cm apart, in line with the screws on the upper shelf, on the lower shelf.

*Test 3 – “Overhead Work”*


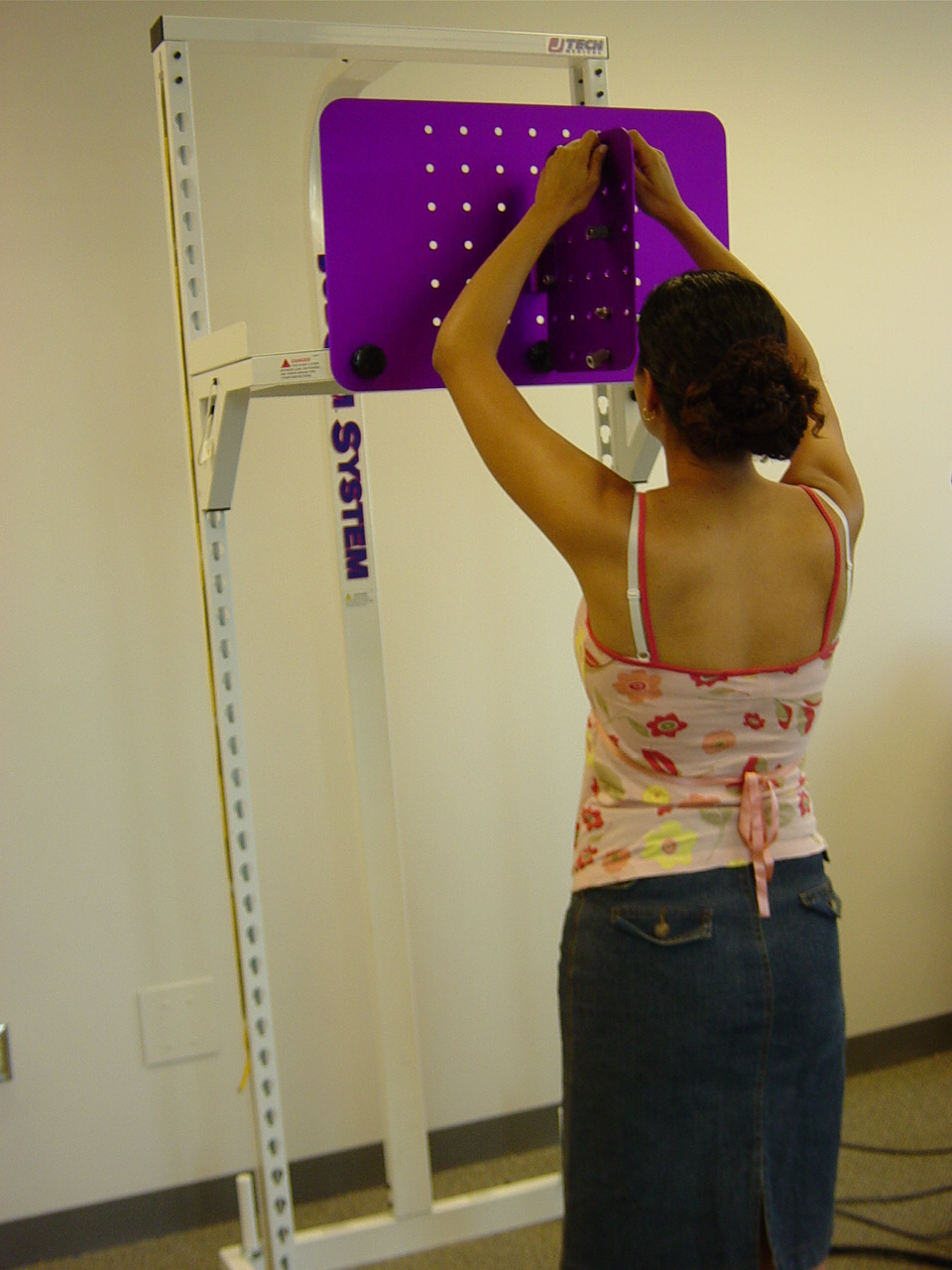


A shelf is placed at the subject’s eye level with an attachable plate, perpendicular to the shelf, projecting out toward the subject.


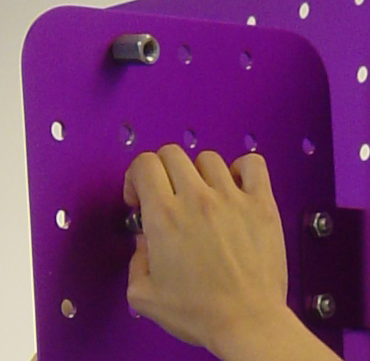


One bolt is placed in the top notch of the attachable plate and a second bolt is placed in the third notch down the same column so that there is an empty notch between them.

The bolts (3/8-16x2/4) are arranged so that the standoff and nuts are on alternating sides (e.g., Bolt 1: standoff on left, nut on right; Bolt 2: standoff on right, nut on left).

**Test Protocol**

- Test 1 – “Waist-Up”: Using the affected arm, the patient lifts three 1-kg containers, one at a time, between a shelf at waist level and a shelf 25 cm higher at a speed of 60 beats per minute, controlled by a metronome (beat 1 - grab, beat 2 – lift and place). Controls are tested using the dominant hand. The subjects and controls perform the task until 5 minutes have elapsed or they feel unable to continue (see test-stopping criteria below).
  - *Order and placement of the containers:* Subjects start with the container right in front of them (e.g., the container closest to the hand to be tested) and lift the distant one last. The first container is lifted to the higher shelf, then the second and third containers. When all the containers are on the higher shelf, the subject returns to the beginning and moves the containers down.
  - *Standing position:* The patient stands with their feet apart, flat on the ground. When their elbow is tucked at their side, the tip of their finger should touch the lower shelf.
- Test 2 – “EYE-DOWN”: Using the affected arm, the patient lifts three 1-kg containers, one at a time, between a shelf at eye level and a shelf 25 cm lower at a speed of 60 beats per minute, controlled by a metronome (beat 1 - grab, beat 2 – lift and place). Controls are tested using the dominant hand. The subjects and controls perform the task until 5 minutes have elapsed or they feel unable to continue (see test-stopping criteria below).
  - *Order and placement of the containers:* Subjects start with the container right in front of them (e.g., the container closest to the hand to be tested) and lift the distant one last. The first container is lifted to the higher shelf, then the second and third containers. When all the containers are on the higher shelf, the subject returns to the beginning and moves the containers down.
  - *Standing position:* The patient stands with their feet apart, flat on the ground. When their elbow is tucked at their side, the tip of their finger should touch the lower shelf.

*Tests 1 and 2 instructions for subjects:*

“We would like you to move all 3 containers from this shelf up/back down following the beat of the metronome (60 beats per minute). If you are off cadence, feel pain, or just simply can't continue, let us know and we will stop the timer. If you have reached 5 minutes, the subtest is over and you can rest before the next test.”

- Test 3 – “Overhead Work”: Using both arms, the subject repeatedly screws and unscrews bolts (the nut is held, while the standoff is turned) in the top 3 holes in the plate, simulating sustained overhead work.
  - *Pattern:* The bolt in notch 1 (top) moves down to notch 2.

The bolt in notch 3 (bottom) moves up to notch 1.

The bolt in notch 2 moves down to notch 3.

This pattern is repeated until 5 minutes have elapsed or the subjects feel unable to continue (see test-stopping criteria below).

- - *Standing position:* The patient stands with their feet apart, flat on the ground. When their hands are held up, the elbows should be bent (start position).

*Test 3 instructions for subjects:*

“Screw and unscrew the bolts by staying in the top 3 holes. We want you to hold the nut and turn the standoff. Do NOT twirl the screw. If you drop a bolt, keep your arms up in the air and a tester will give you another one so that you don't bring your arms down.” (the tester always has one or two extra bolts ready to go).

*Test Stopping Criteria*

Each task can be continued for up to 5 minutes, but is terminated based on the following stopping rules:

1. The subject stops or states it is too painful to continue.
2. The subject is severely off pacing to the extent that they are unable to complete one repetition of the movement within 2 beats of the metronome.
3. The subject substitutes using trunk/whole body movement and cannot correct with feedback for 5 successive repetitions of the task.
4. The examiner believes the subject is at risk of injury or adverse complication if tests were to continue.

- There is an approximately 30-sec rest in between tests as the shelves are adjusted and the patient resumes start position.
- **Scoring:** The times are measured using a stopwatch.
  - - Test 1 (Waist-Up)/300 sec X 100%
    - Test 2 (Eye-Down)/ 300 sec X 100%
    - Test 3 (Overhead Work)/300 sec X 100%
    - Total Score = Mean of Test 1, Test 2 and Test 3

**Comparative Data**

| **Population** | **Sex** | **n** | **Test 1**  **Score in sec (SD), %** | **Test 2**  **Score in sec (SD), %** | **Test 3**  **Score in sec (SD), %** | **Total**  **Score in sec (SD), %** |
| --- | --- | --- | --- | --- | --- | --- |
| **Controls** |  |  |  |  |  |  |
| Development study | M, F | 5 | 300.00  100% | 286.50  95.50% | 300.00  100% | 295.50  98.50% |
| Validation study | M | 8 | 300.00 (.00)  100% | 276.50  (35.97)  92.16% | 300.00 (.00)  100% | 292.16 (11.98)  97.38% |
| Validation study | F | 11 | 300.00 (.00)  100% | 299.09 (3.02)  99.69% | 300.00 (.00)  100% | 299.69  (1.00)  99.89% |
| **Patients** |  |  |  |  |  |  |
| Development study | M, F | 5 | 178.80  59.60% | 116.60  38.87% | 150.70  50.23% | 148.70  49.57% |
| Validation study | M | 8 | 300.00 (.00)  100% | 246.25 (67.30)  82.08% | 278.75 (60.10)  92.91% | 275.00 (24.78)  91.66% |
| Validation study | F | 9 | 300.00 (.00)  100% | 246.00 (83.90)  82.00% | 271.22 (44.96)  90.40% | 272.40 (42.60)  90.80% |

Legend: F = female, M = male

**Acknowledgements**

Investigation of the impact of reach and grasp activities on aspect of EMG and kinematics were formed in the Human Movement Laboratory at McMaster University (Principal investigator – V Galea). The original pilot testing and test development was conducted with D Humphreys, J McCluskie and E Shewayhat. Further development and validation of the test in mild shoulder pathology was performed by M Ghobrial, KB Quirion, M St-Amour, T Tsui. The wooden shelving unit was built by James Bromley.

**Appendix 1: Wooden Shelving Unit**


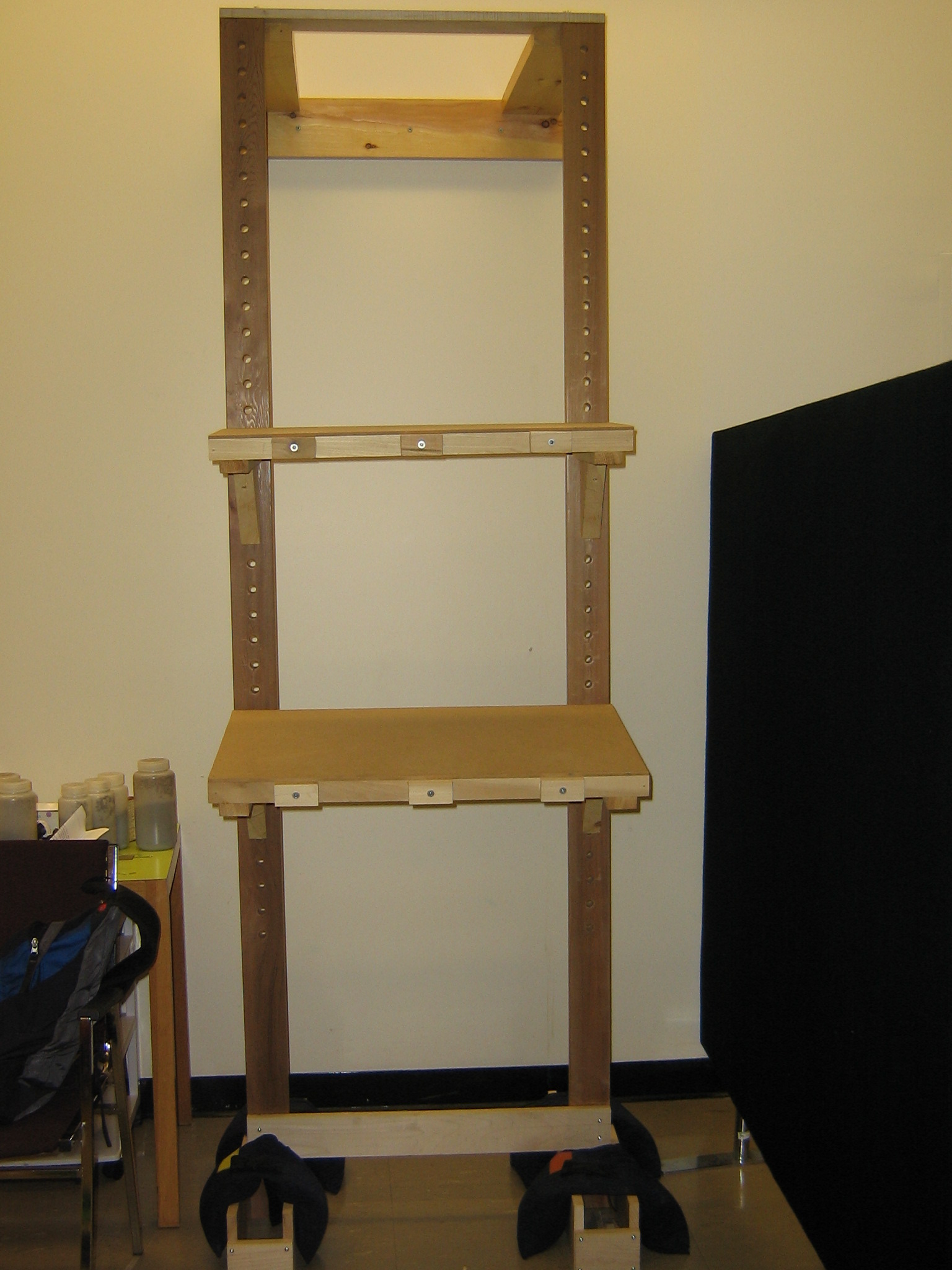


An adjustable shelving unit was constructed using self-made material. The unit consisted of two cedar uprights with dimensions 3.5 cm X 8.5 cm X 236.5 cm. Holes (2.5 cm diameter) were drilled into the uprights with a 5 cm center to center distance. Two shelves were constructed (78 cm X 45 cm) and were adjustable via two posts made out of 2.5 cm thick dowels. One shelf was constructed with additional objects allowing a dexterity task to be performed with arms raised above the head. The task consisted of fitting turn screws into 6 fittings. The present prototype is not free-standing. The two uprights were fixed into the wall via cedar boards and rested on cedar board frame that was weighted down with sandbags. We are presently adapting this prototype to a free-standing unit.
